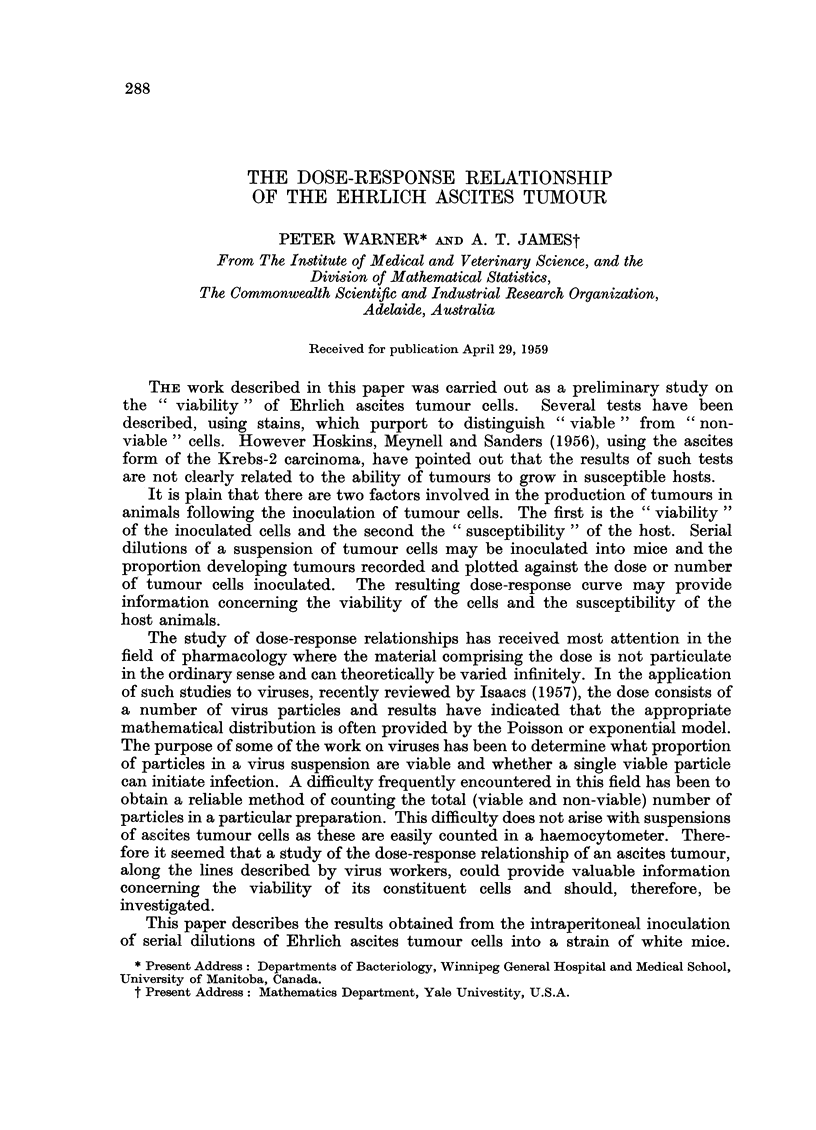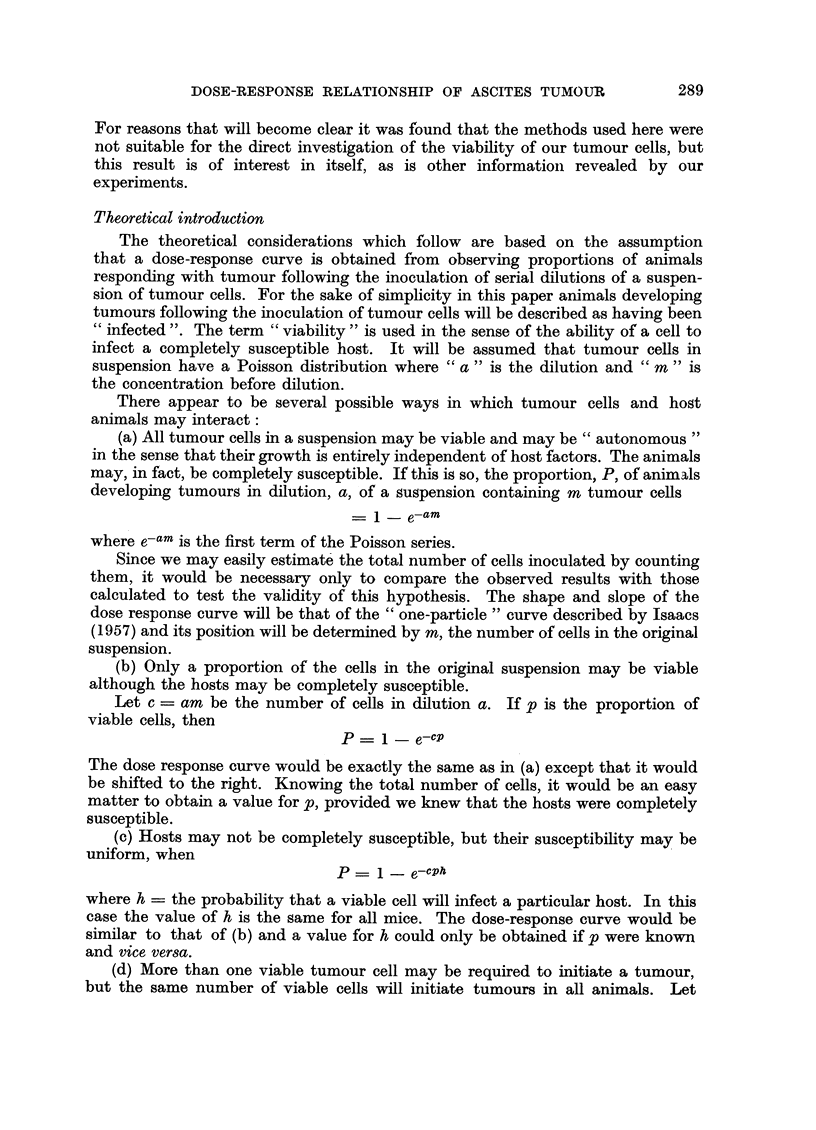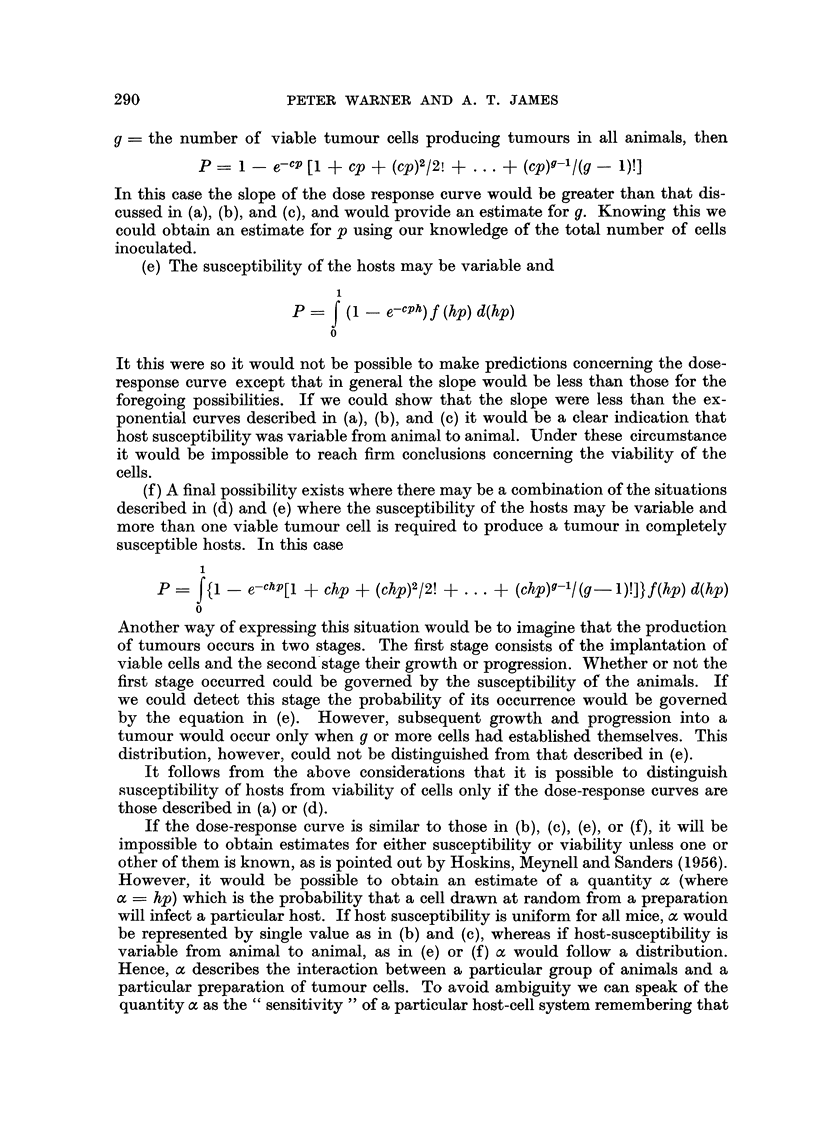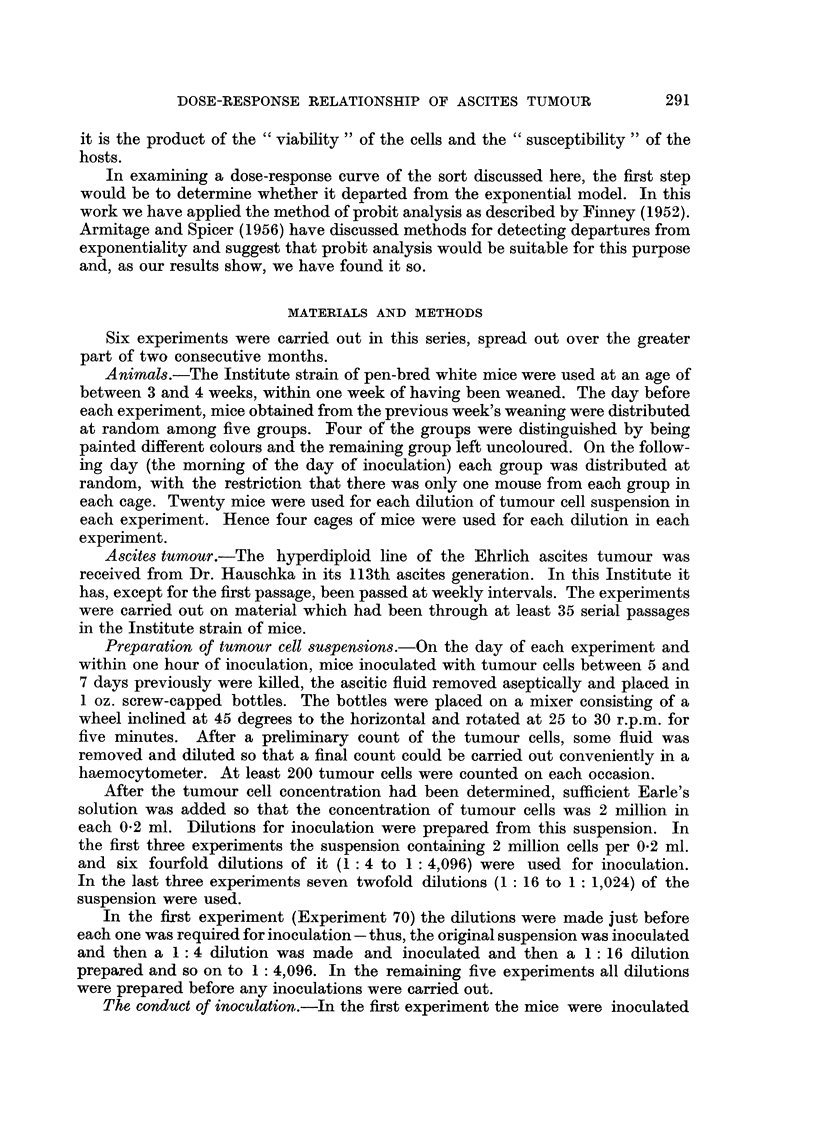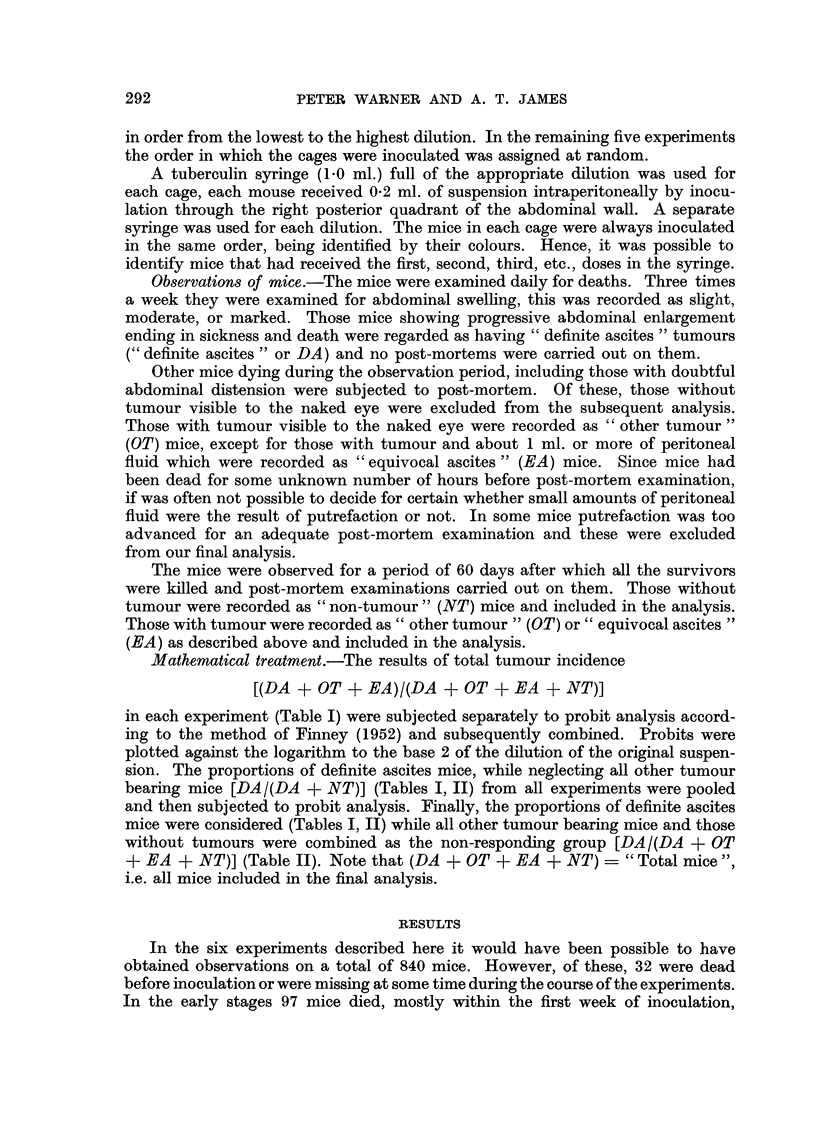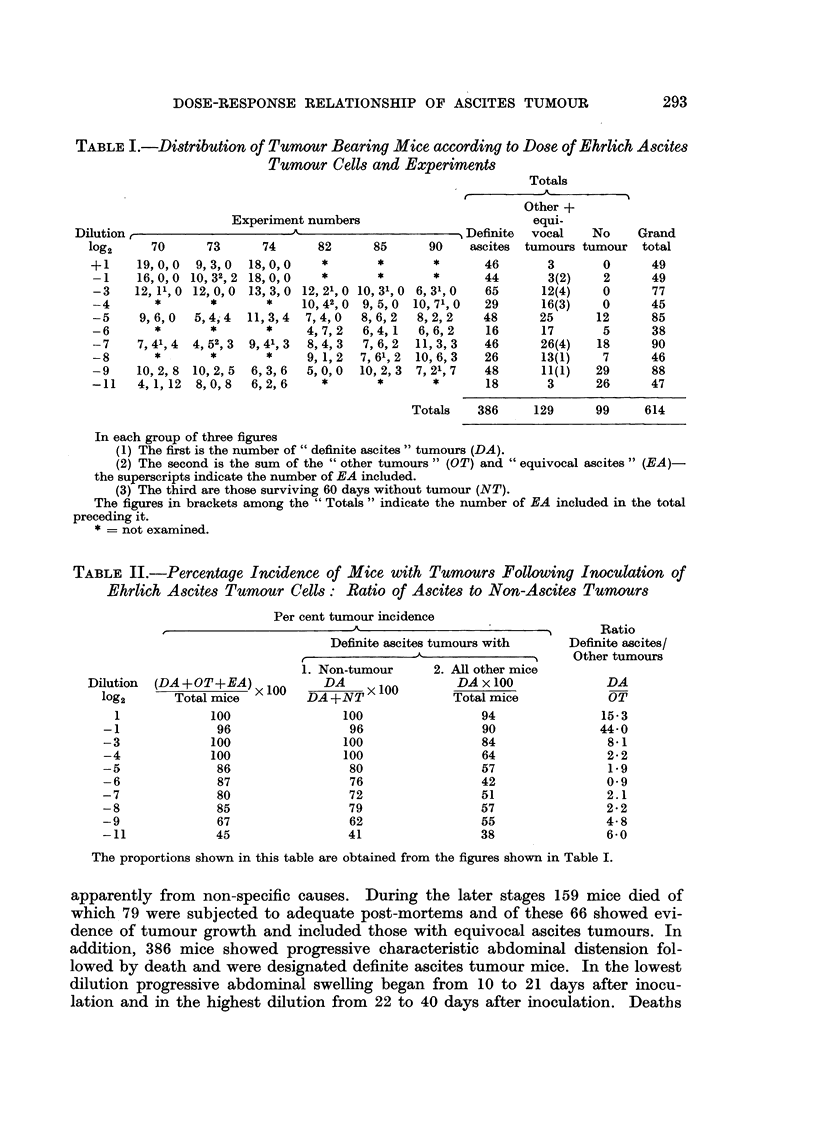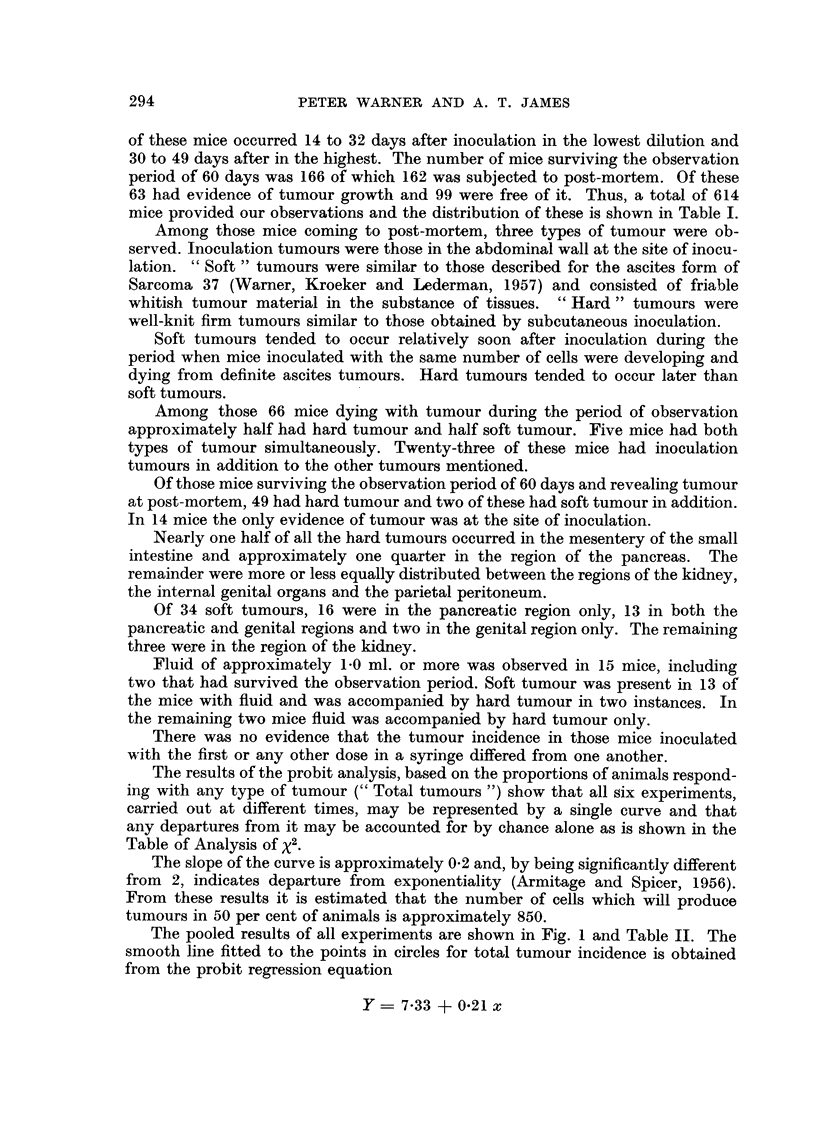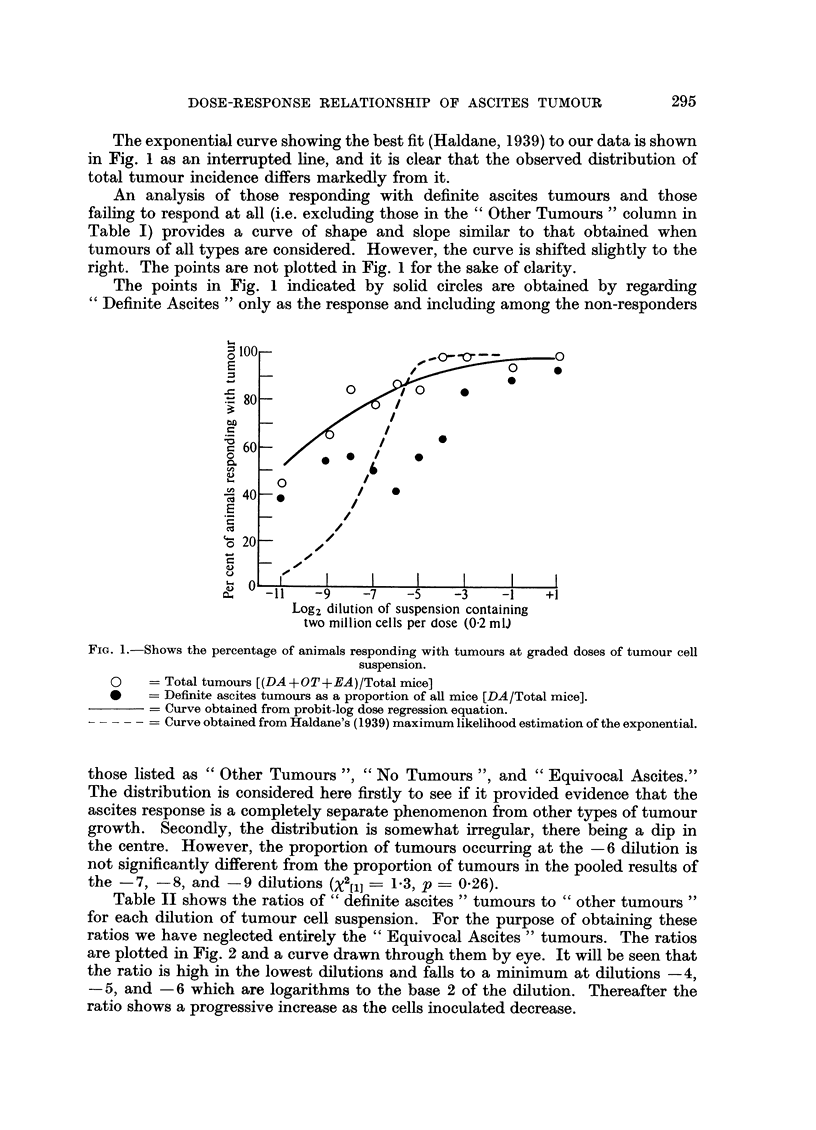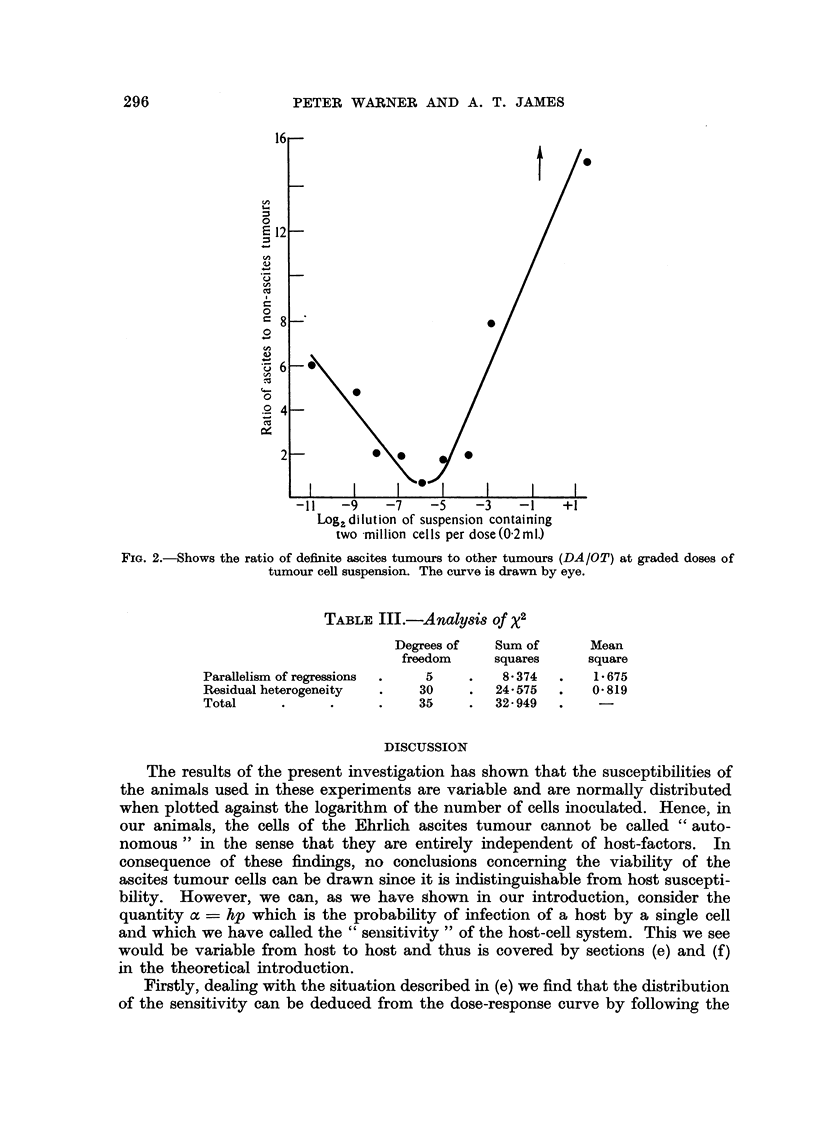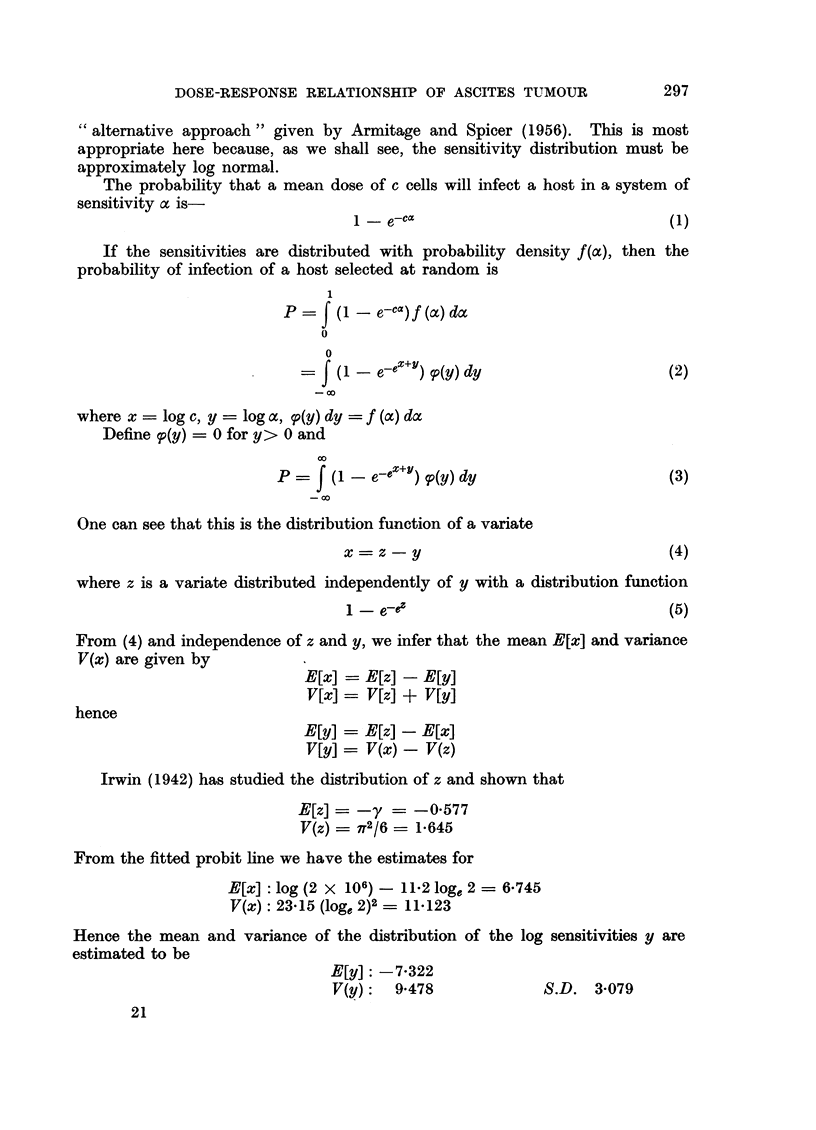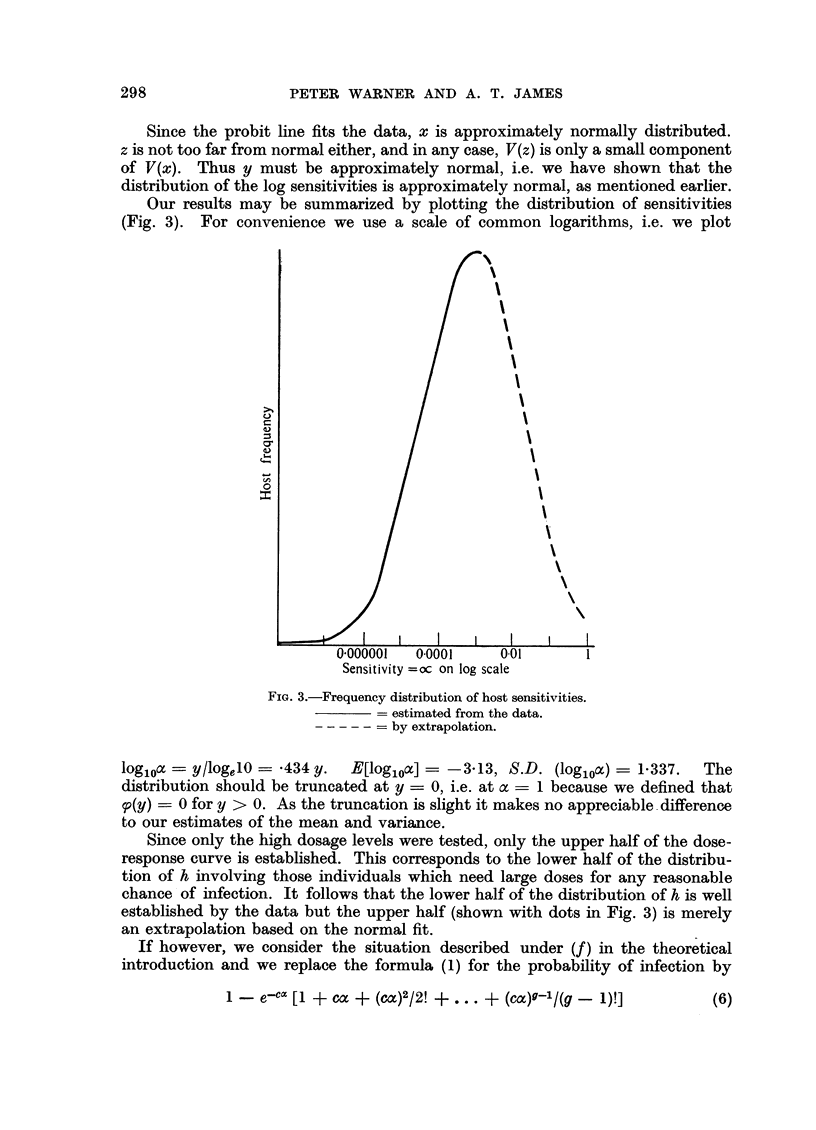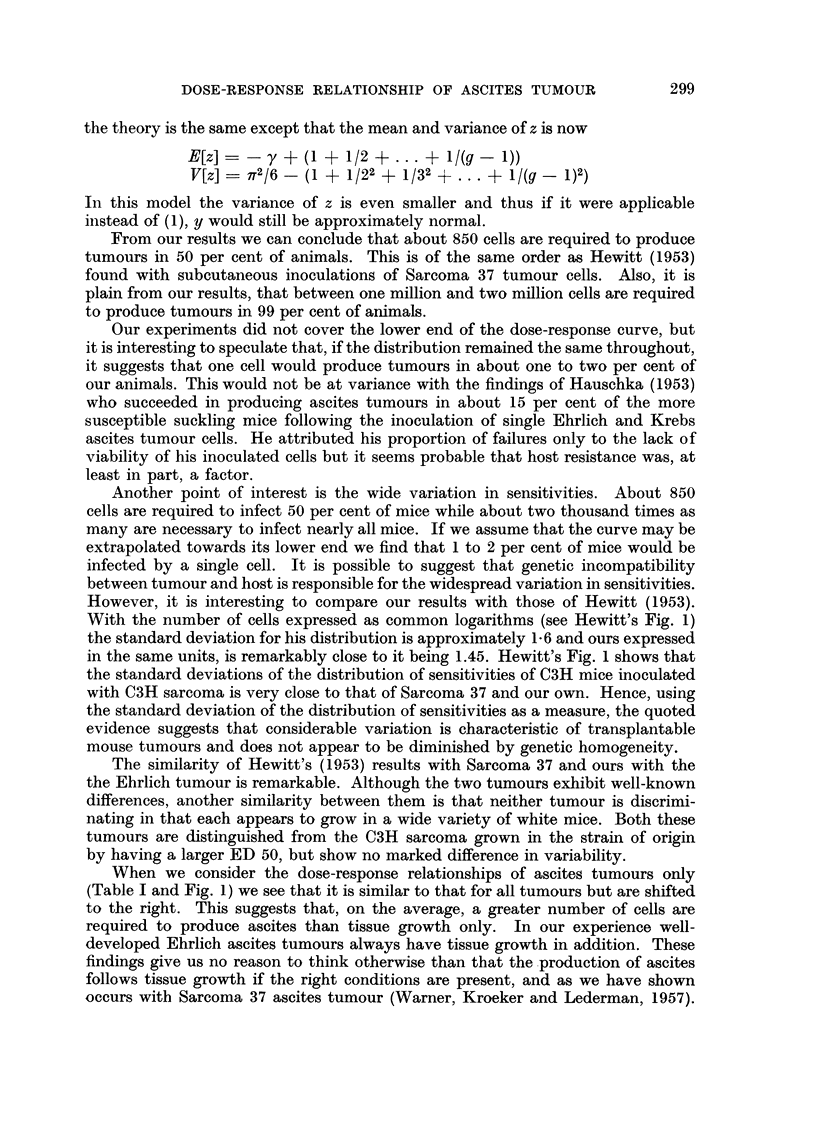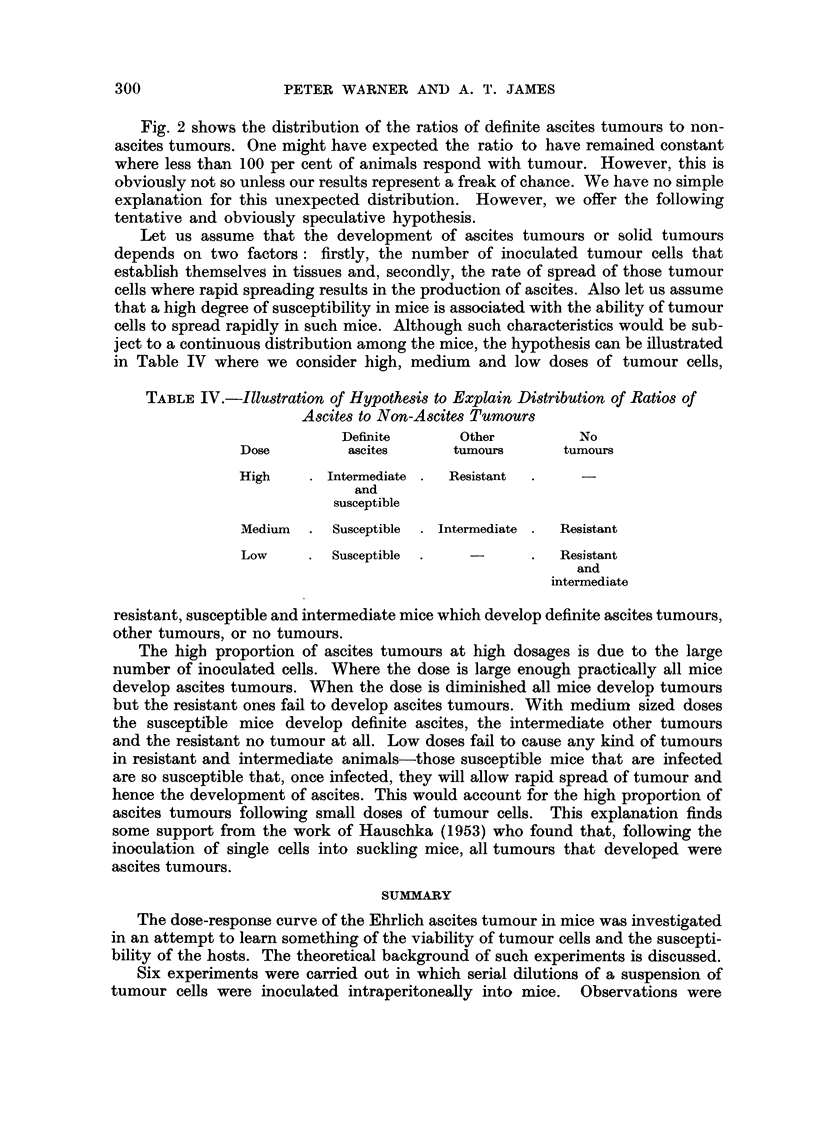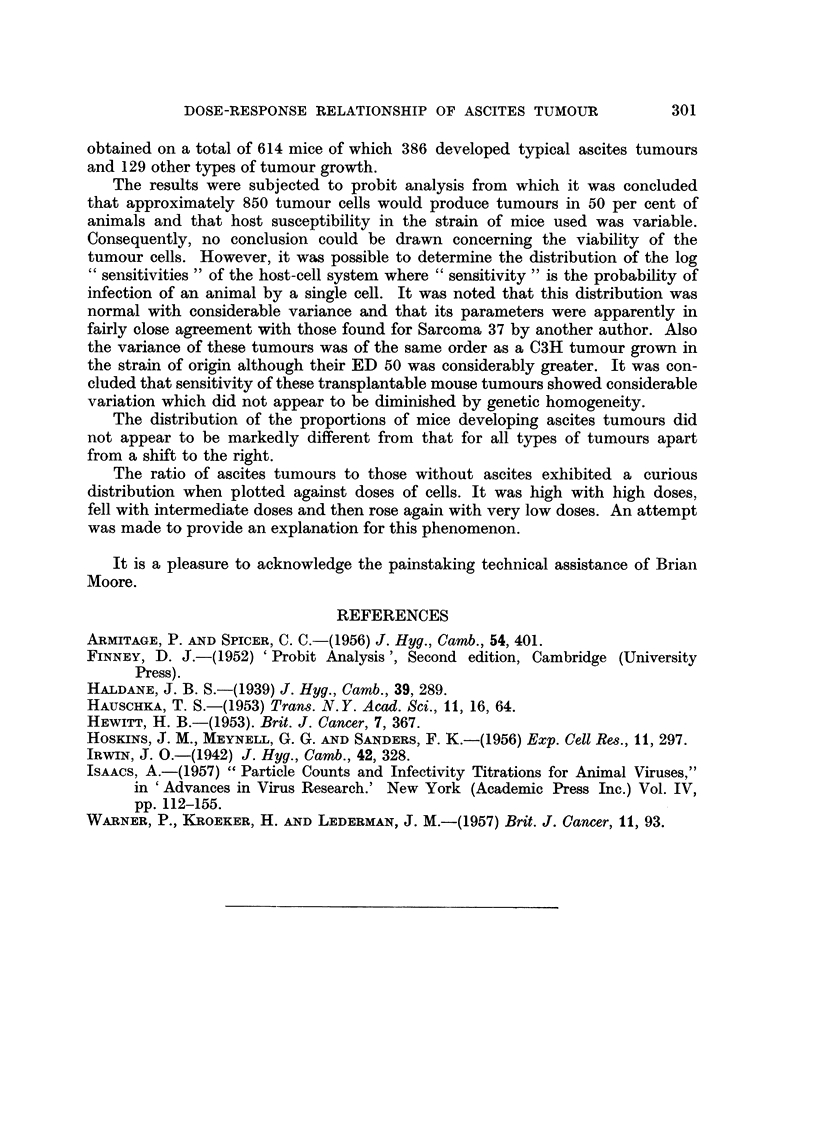# The Dose-response Relationship of the Ehrlich Ascites Tumour

**DOI:** 10.1038/bjc.1959.35

**Published:** 1959-06

**Authors:** Peter Warner, A. T. James


### THE DOSE-RESPONSE RELATIONSHIP OF THE EHRLICH ASCITES TUMOUR

PETER WARNER* AND A. T. JAMES†

*From The Institute of Medical and Veterinary Science, and the Division of Mathematical Statistics, The Commonwealth Scientific and Industrial Research Organization, Adelaide, Australia*

Received for publication April 29, 1959

THE work described in this paper was carried out as a preliminary study on the " viability " of Ehrlich ascites tumour cells. Several tests have been described, using stains, which purport to distinguish " viable " from " non-viable " cells. However Hoskins, Meynell and Sanders (1956), using the ascites form of the Krebs-2 carcinoma, have pointed out that the results of such tests are not clearly related to the ability of tumours to grow in susceptible hosts.

It is plain that there are two factors involved in the production of tumours in animals following the inoculation of tumour cells. The first is the " viability " of the inoculated cells and the second the " susceptibility " of the host. Serial dilutions of a suspension of tumour cells may be inoculated into mice and the proportion developing tumours recorded and plotted against the dose or number of tumour cells inoculated. The resulting dose-response curve may provide information concerning the viability of the cells and the susceptibility of the host animals.

The study of dose-response relationships has received most attention in the field of pharmacology where the material comprising the dose is not particulate in the ordinary sense and can theoretically be varied infinitely. In the application of such studies to viruses, recently reviewed by Isaacs (1957), the dose consists of a number of virus particles and results have indicated that the appropriate mathematical distribution is often provided by the Poisson or exponential model. The purpose of some of the work on viruses has been to determine what proportion of particles in a virus suspension are viable and whether a single viable particle can initiate infection. A difficulty frequently encountered in this field has been to obtain a reliable method of counting the total (viable and non-viable) number of particles in a particular preparation. This difficulty does not arise with suspensions of ascites tumour cells as these are easily counted in a haemocytometer. Therefore it seemed that a study of the dose-response relationship of an ascites tumour, along the lines described by virus workers, could provide valuable information concerning the viability of its constituent cells and should, therefore, be investigated.

This paper describes the results obtained from the intraperitoneal inoculation of serial dilutions of Ehrlich ascites tumour cells into a strain of white mice.

* Present Address : Departments of Bacteriology, Winnipeg General Hospital and Medical School, University of Manitoba, Canada.

† Present Address : Mathematics Department, Yale Univestity, U.S.A.

For reasons that will become clear it was found that the methods used here were not suitable for the direct investigation of the viability of our tumour cells, but this result is of interest in itself, as is other information revealed by our experiments.

*Theoretical introduction*

The theoretical considerations which follow are based on the assumption that a dose-response curve is obtained from observing proportions of animals responding with tumour following the inoculation of serial dilutions of a suspension of tumour cells. For the sake of simplicity in this paper animals developing tumours following the inoculation of tumour cells will be described as having been "infected". The term "viability" is used in the sense of the ability of a cell to infect a completely susceptible host. It will be assumed that tumour cells in suspension have a Poisson distribution where "$a$" is the dilution and "$m$" is the concentration before dilution.

There appear to be several possible ways in which tumour cells and host animals may interact:

(a) All tumour cells in a suspension may be viable and may be "autonomous" in the sense that their growth is entirely independent of host factors. The animals may, in fact, be completely susceptible. If this is so, the proportion, $P$, of animals developing tumours in dilution, $a$, of a suspension containing $m$ tumour cells

$$= 1 - e^{-am}$$

where $e^{-am}$ is the first term of the Poisson series.

Since we may easily estimate the total number of cells inoculated by counting them, it would be necessary only to compare the observed results with those calculated to test the validity of this hypothesis. The shape and slope of the dose response curve will be that of the "one-particle" curve described by Isaacs (1957) and its position will be determined by $m$, the number of cells in the original suspension.

(b) Only a proportion of the cells in the original suspension may be viable although the hosts may be completely susceptible.

Let $c = am$ be the number of cells in dilution $a$. If $p$ is the proportion of viable cells, then

$$P = 1 - e^{-cp}$$

The dose response curve would be exactly the same as in (a) except that it would be shifted to the right. Knowing the total number of cells, it would be an easy matter to obtain a value for $p$, provided we knew that the hosts were completely susceptible.

(c) Hosts may not be completely susceptible, but their susceptibility may be uniform, when

$$P = 1 - e^{-cph}$$

where $h$ = the probability that a viable cell will infect a particular host. In this case the value of $h$ is the same for all mice. The dose-response curve would be similar to that of (b) and a value for $h$ could only be obtained if $p$ were known and *vice versa*.

(d) More than one viable tumour cell may be required to initiate a tumour, but the same number of viable cells will initiate tumours in all animals. Let

$g$ = the number of viable tumour cells producing tumours in all animals, then

$$P = 1 - e^{-cp}[1 + cp + (cp)^2/2! + \ldots + (cp)^{g-1}/(g-1)!]$$

In this case the slope of the dose response curve would be greater than that discussed in (a), (b), and (c), and would provide an estimate for $g$. Knowing this we could obtain an estimate for $p$ using our knowledge of the total number of cells inoculated.

(e) The susceptibility of the hosts may be variable and

$$P = \int_0^1 (1 - e^{-cph}) f(hp)\, d(hp)$$

It this were so it would not be possible to make predictions concerning the dose-response curve except that in general the slope would be less than those for the foregoing possibilities. If we could show that the slope were less than the exponential curves described in (a), (b), and (c) it would be a clear indication that host susceptibility was variable from animal to animal. Under these circumstance it would be impossible to reach firm conclusions concerning the viability of the cells.

(f) A final possibility exists where there may be a combination of the situations described in (d) and (e) where the susceptibility of the hosts may be variable and more than one viable tumour cell is required to produce a tumour in completely susceptible hosts. In this case

$$P = \int_0^1 \{1 - e^{-chp}[1 + chp + (chp)^2/2! + \ldots + (chp)^{g-1}/(g-1)!]\} f(hp)\, d(hp)$$

Another way of expressing this situation would be to imagine that the production of tumours occurs in two stages. The first stage consists of the implantation of viable cells and the second stage their growth or progression. Whether or not the first stage occurred could be governed by the susceptibility of the animals. If we could detect this stage the probability of its occurrence would be governed by the equation in (e). However, subsequent growth and progression into a tumour would occur only when $g$ or more cells had established themselves. This distribution, however, could not be distinguished from that described in (e).

It follows from the above considerations that it is possible to distinguish susceptibility of hosts from viability of cells only if the dose-response curves are those described in (a) or (d).

If the dose-response curve is similar to those in (b), (c), (e), or (f), it will be impossible to obtain estimates for either susceptibility or viability unless one or other of them is known, as is pointed out by Hoskins, Meynell and Sanders (1956). However, it would be possible to obtain an estimate of a quantity $\alpha$ (where $\alpha = hp$) which is the probability that a cell drawn at random from a preparation will infect a particular host. If host susceptibility is uniform for all mice, $\alpha$ would be represented by single value as in (b) and (c), whereas if host-susceptibility is variable from animal to animal, as in (e) or (f) $\alpha$ would follow a distribution. Hence, $\alpha$ describes the interaction between a particular group of animals and a particular preparation of tumour cells. To avoid ambiguity we can speak of the quantity $\alpha$ as the " sensitivity " of a particular host-cell system remembering that it is the product of the " viability " of the cells and the " susceptibility " of the hosts.

In examining a dose-response curve of the sort discussed here, the first step would be to determine whether it departed from the exponential model. In this work we have applied the method of probit analysis as described by Finney (1952). Armitage and Spicer (1956) have discussed methods for detecting departures from exponentiality and suggest that probit analysis would be suitable for this purpose and, as our results show, we have found it so.

### MATERIALS AND METHODS

Six experiments were carried out in this series, spread out over the greater part of two consecutive months.

*Animals.*—The Institute strain of pen-bred white mice were used at an age of between 3 and 4 weeks, within one week of having been weaned. The day before each experiment, mice obtained from the previous week's weaning were distributed at random among five groups. Four of the groups were distinguished by being painted different colours and the remaining group left uncoloured. On the following day (the morning of the day of inoculation) each group was distributed at random, with the restriction that there was only one mouse from each group in each cage. Twenty mice were used for each dilution of tumour cell suspension in each experiment. Hence four cages of mice were used for each dilution in each experiment.

*Ascites tumour.*—The hyperdiploid line of the Ehrlich ascites tumour was received from Dr. Hauschka in its 113th ascites generation. In this Institute it has, except for the first passage, been passed at weekly intervals. The experiments were carried out on material which had been through at least 35 serial passages in the Institute strain of mice.

*Preparation of tumour cell suspensions.*—On the day of each experiment and within one hour of inoculation, mice inoculated with tumour cells between 5 and 7 days previously were killed, the ascitic fluid removed aseptically and placed in 1 oz. screw-capped bottles. The bottles were placed on a mixer consisting of a wheel inclined at 45 degrees to the horizontal and rotated at 25 to 30 r.p.m. for five minutes. After a preliminary count of the tumour cells, some fluid was removed and diluted so that a final count could be carried out conveniently in a haemocytometer. At least 200 tumour cells were counted on each occasion.

After the tumour cell concentration had been determined, sufficient Earle's solution was added so that the concentration of tumour cells was 2 million in each 0·2 ml. Dilutions for inoculation were prepared from this suspension. In the first three experiments the suspension containing 2 million cells per 0·2 ml. and six fourfold dilutions of it (1 : 4 to 1 : 4,096) were used for inoculation. In the last three experiments seven twofold dilutions (1 : 16 to 1 : 1,024) of the suspension were used.

In the first experiment (Experiment 70) the dilutions were made just before each one was required for inoculation – thus, the original suspension was inoculated and then a 1 : 4 dilution was made and inoculated and then a 1 : 16 dilution prepared and so on to 1 : 4,096. In the remaining five experiments all dilutions were prepared before any inoculations were carried out.

*The conduct of inoculation.*—In the first experiment the mice were inoculated in order from the lowest to the highest dilution. In the remaining five experiments the order in which the cages were inoculated was assigned at random.

A tuberculin syringe (1·0 ml.) full of the appropriate dilution was used for each cage, each mouse received 0·2 ml. of suspension intraperitoneally by inoculation through the right posterior quadrant of the abdominal wall. A separate syringe was used for each dilution. The mice in each cage were always inoculated in the same order, being identified by their colours. Hence, it was possible to identify mice that had received the first, second, third, etc., doses in the syringe.

*Observations of mice.*—The mice were examined daily for deaths. Three times a week they were examined for abdominal swelling, this was recorded as slight, moderate, or marked. Those mice showing progressive abdominal enlargement ending in sickness and death were regarded as having " definite ascites " tumours (" definite ascites " or *DA*) and no post-mortems were carried out on them.

Other mice dying during the observation period, including those with doubtful abdominal distension were subjected to post-mortem. Of these, those without tumour visible to the naked eye were excluded from the subsequent analysis. Those with tumour visible to the naked eye were recorded as " other tumour " (*OT*) mice, except for those with tumour and about 1 ml. or more of peritoneal fluid which were recorded as " equivocal ascites " (*EA*) mice. Since mice had been dead for some unknown number of hours before post-mortem examination, if was often not possible to decide for certain whether small amounts of peritoneal fluid were the result of putrefaction or not. In some mice putrefaction was too advanced for an adequate post-mortem examination and these were excluded from our final analysis.

The mice were observed for a period of 60 days after which all the survivors were killed and post-mortem examinations carried out on them. Those without tumour were recorded as " non-tumour " (*NT*) mice and included in the analysis. Those with tumour were recorded as " other tumour " (*OT*) or " equivocal ascites " (*EA*) as described above and included in the analysis.

*Mathematical treatment.*—The results of total tumour incidence

$$[(DA + OT + EA)/(DA + OT + EA + NT)]$$

in each experiment (Table I) were subjected separately to probit analysis according to the method of Finney (1952) and subsequently combined. Probits were plotted against the logarithm to the base 2 of the dilution of the original suspension. The proportions of definite ascites mice, while neglecting all other tumour bearing mice $[DA/(DA + NT)]$ (Tables I, II) from all experiments were pooled and then subjected to probit analysis. Finally, the proportions of definite ascites mice were considered (Tables I, II) while all other tumour bearing mice and those without tumours were combined as the non-responding group $[DA/(DA + OT + EA + NT)]$ (Table II). Note that $(DA + OT + EA + NT) =$ " Total mice ", i.e. all mice included in the final analysis.

### RESULTS

In the six experiments described here it would have been possible to have obtained observations on a total of 840 mice. However, of these, 32 were dead before inoculation or were missing at some time during the course of the experiments. In the early stages 97 mice died, mostly within the first week of inoculation,

TABLE I.—*Distribution of Tumour Bearing Mice according to Dose of Ehrlich Ascites Tumour Cells and Experiments*

| Dilution $\log_2$ | Experiment numbers 70 | 73 | 74 | 82 | 85 | 90 | Totals Definite ascites | Other + equivocal tumours | No tumour | Grand total |
|---|---|---|---|---|---|---|---|---|---|---|
| +1 | 19, 0, 0 | 9, 3, 0 | 18, 0, 0 | * | * | * | 46 | 3 | 0 | 49 |
| −1 | 16, 0, 0 | 10, $3^{2}$, 2 | 18, 0, 0 | * | * | * | 44 | 3(2) | 2 | 49 |
| −3 | 12, $1^{1}$, 0 | 12, 0, 0 | 13, 3, 0 | 12, $2^{1}$, 0 | 10, $3^{1}$, 0 | 6, $3^{1}$, 0 | 65 | 12(4) | 0 | 77 |
| −4 | * | * | * | 10, $4^{2}$, 0 | 9, 5, 0 | 10, $7^{1}$, 0 | 29 | 16(3) | 0 | 45 |
| −5 | 9, 6, 0 | 5, 4, 4 | 11, 3, 4 | 7, 4, 0 | 8, 6, 2 | 8, 2, 2 | 48 | 25 | 12 | 85 |
| −6 | * | * | * | 4, 7, 2 | 6, 4, 1 | 6, 6, 2 | 16 | 17 | 5 | 38 |
| −7 | 7, $4^{1}$, 4 | 4, $5^{2}$, 3 | 9, $4^{1}$, 3 | 8, 4, 3 | 7, 6, 2 | 11, 3, 3 | 46 | 26(4) | 18 | 90 |
| −8 | * | * | * | 9, 1, 2 | 7, $6^{1}$, 2 | 10, 6, 3 | 26 | 13(1) | 7 | 46 |
| −9 | 10, 2, 8 | 10, 2, 5 | 6, 3, 6 | 5, 0, 0 | 10, 2, 3 | 7, $2^{1}$, 7 | 48 | 11(1) | 29 | 88 |
| −11 | 4, 1, 12 | 8, 0, 8 | 6, 2, 6 | * | * | * | 18 | 3 | 26 | 47 |
| | | | | | | Totals | 386 | 129 | 99 | 614 |

In each group of three figures

(1) The first is the number of " definite ascites " tumours (*DA*).

(2) The second is the sum of the " other tumours " (*OT*) and " equivocal ascites " (*EA*)—the superscripts indicate the number of *EA* included.

(3) The third are those surviving 60 days without tumour (*NT*).

The figures in brackets among the " Totals " indicate the number of *EA* included in the total preceding it.

* = not examined.

TABLE II.—*Percentage Incidence of Mice with Tumours Following Inoculation of Ehrlich Ascites Tumour Cells : Ratio of Ascites to Non-Ascites Tumours*

| Dilution $\log_2$ | Per cent tumour incidence $\frac{(DA+OT+EA)}{\text{Total mice}}\times 100$ | Definite ascites tumours with 1. Non-tumour $\frac{DA}{DA+NT}\times 100$ | 2. All other mice $\frac{DA\times 100}{\text{Total mice}}$ | Ratio Definite ascites/ Other tumours $\frac{DA}{OT}$ |
|---|---|---|---|---|
| 1 | 100 | 100 | 94 | 15·3 |
| −1 | 96 | 96 | 90 | 44·0 |
| −3 | 100 | 100 | 84 | 8·1 |
| −4 | 100 | 100 | 64 | 2·2 |
| −5 | 86 | 80 | 57 | 1·9 |
| −6 | 87 | 76 | 42 | 0·9 |
| −7 | 80 | 72 | 51 | 2.1 |
| −8 | 85 | 79 | 57 | 2·2 |
| −9 | 67 | 62 | 55 | 4·8 |
| −11 | 45 | 41 | 38 | 6·0 |

The proportions shown in this table are obtained from the figures shown in Table I.

apparently from non-specific causes. During the later stages 159 mice died of which 79 were subjected to adequate post-mortems and of these 66 showed evidence of tumour growth and included those with equivocal ascites tumours. In addition, 386 mice showed progressive characteristic abdominal distension followed by death and were designated definite ascites tumour mice. In the lowest dilution progressive abdominal swelling began from 10 to 21 days after inoculation and in the highest dilution from 22 to 40 days after inoculation. Deaths of these mice occurred 14 to 32 days after inoculation in the lowest dilution and 30 to 49 days after in the highest. The number of mice surviving the observation period of 60 days was 166 of which 162 was subjected to post-mortem. Of these 63 had evidence of tumour growth and 99 were free of it. Thus, a total of 614 mice provided our observations and the distribution of these is shown in Table I.

Among those mice coming to post-mortem, three types of tumour were observed. Inoculation tumours were those in the abdominal wall at the site of inoculation. " Soft " tumours were similar to those described for the ascites form of Sarcoma 37 (Warner, Kroeker and Lederman, 1957) and consisted of friable whitish tumour material in the substance of tissues. " Hard " tumours were well-knit firm tumours similar to those obtained by subcutaneous inoculation.

Soft tumours tended to occur relatively soon after inoculation during the period when mice inoculated with the same number of cells were developing and dying from definite ascites tumours. Hard tumours tended to occur later than soft tumours.

Among those 66 mice dying with tumour during the period of observation approximately half had hard tumour and half soft tumour. Five mice had both types of tumour simultaneously. Twenty-three of these mice had inoculation tumours in addition to the other tumours mentioned.

Of those mice surviving the observation period of 60 days and revealing tumour at post-mortem, 49 had hard tumour and two of these had soft tumour in addition. In 14 mice the only evidence of tumour was at the site of inoculation.

Nearly one half of all the hard tumours occurred in the mesentery of the small intestine and approximately one quarter in the region of the pancreas. The remainder were more or less equally distributed between the regions of the kidney, the internal genital organs and the parietal peritoneum.

Of 34 soft tumours, 16 were in the pancreatic region only, 13 in both the pancreatic and genital regions and two in the genital region only. The remaining three were in the region of the kidney.

Fluid of approximately 1·0 ml. or more was observed in 15 mice, including two that had survived the observation period. Soft tumour was present in 13 of the mice with fluid and was accompanied by hard tumour in two instances. In the remaining two mice fluid was accompanied by hard tumour only.

There was no evidence that the tumour incidence in those mice inoculated with the first or any other dose in a syringe differed from one another.

The results of the probit analysis, based on the proportions of animals responding with any type of tumour (" Total tumours ") show that all six experiments, carried out at different times, may be represented by a single curve and that any departures from it may be accounted for by chance alone as is shown in the Table of Analysis of $\chi^2$.

The slope of the curve is approximately 0·2 and, by being significantly different from 2, indicates departure from exponentiality (Armitage and Spicer, 1956). From these results it is estimated that the number of cells which will produce tumours in 50 per cent of animals is approximately 850.

The pooled results of all experiments are shown in Fig. 1 and Table II. The smooth line fitted to the points in circles for total tumour incidence is obtained from the probit regression equation

$$Y = 7{\cdot}33 + 0{\cdot}21\,x$$

The exponential curve showing the best fit (Haldane, 1939) to our data is shown in Fig. 1 as an interrupted line, and it is clear that the observed distribution of total tumour incidence differs markedly from it.

An analysis of those responding with definite ascites tumours and those failing to respond at all (i.e. excluding those in the " Other Tumours " column in Table I) provides a curve of shape and slope similar to that obtained when tumours of all types are considered. However, the curve is shifted slightly to the right. The points are not plotted in Fig. 1 for the sake of clarity.

The points in Fig. 1 indicated by solid circles are obtained by regarding " Definite Ascites " only as the response and including among the non-responders

FIG. 1.—Shows the percentage of animals responding with tumours at graded doses of tumour cell suspension.

○ = Total tumours [$(DA+OT+EA)$/Total mice]
● = Definite ascites tumours as a proportion of all mice [$DA$/Total mice].
——— = Curve obtained from probit-log dose regression equation.
– – – – – – = Curve obtained from Haldane's (1939) maximum likelihood estimation of the exponential.

those listed as " Other Tumours ", " No Tumours ", and " Equivocal Ascites." The distribution is considered here firstly to see if it provided evidence that the ascites response is a completely separate phenomenon from other types of tumour growth. Secondly, the distribution is somewhat irregular, there being a dip in the centre. However, the proportion of tumours occurring at the $-6$ dilution is not significantly different from the proportion of tumours in the pooled results of the $-7$, $-8$, and $-9$ dilutions ($\chi^2_{[1]} = 1{\cdot}3$, $p = 0{\cdot}26$).

Table II shows the ratios of " definite ascites " tumours to " other tumours " for each dilution of tumour cell suspension. For the purpose of obtaining these ratios we have neglected entirely the " Equivocal Ascites " tumours. The ratios are plotted in Fig. 2 and a curve drawn through them by eye. It will be seen that the ratio is high in the lowest dilutions and falls to a minimum at dilutions $-4$, $-5$, and $-6$ which are logarithms to the base 2 of the dilution. Thereafter the ratio shows a progressive increase as the cells inoculated decrease.

FIG. 2.—Shows the ratio of definite ascites tumours to other tumours (*DA/OT*) at graded doses of tumour cell suspension. The curve is drawn by eye.

TABLE III.—*Analysis of* $\chi^2$

| | Degrees of freedom | Sum of squares | Mean square |
|---|---|---|---|
| Parallelism of regressions | 5 | 8·374 | 1·675 |
| Residual heterogeneity | 30 | 24·575 | 0·819 |
| Total | 35 | 32·949 | — |

### DISCUSSION

The results of the present investigation has shown that the susceptibilities of the animals used in these experiments are variable and are normally distributed when plotted against the logarithm of the number of cells inoculated. Hence, in our animals, the cells of the Ehrlich ascites tumour cannot be called " autonomous " in the sense that they are entirely independent of host-factors. In consequence of these findings, no conclusions concerning the viability of the ascites tumour cells can be drawn since it is indistinguishable from host susceptibility. However, we can, as we have shown in our introduction, consider the quantity $\alpha = hp$ which is the probability of infection of a host by a single cell and which we have called the " sensitivity " of the host-cell system. This we see would be variable from host to host and thus is covered by sections (e) and (f) in the theoretical introduction.

Firstly, dealing with the situation described in (e) we find that the distribution of the sensitivity can be deduced from the dose-response curve by following the

" alternative approach " given by Armitage and Spicer (1956). This is most appropriate here because, as we shall see, the sensitivity distribution must be approximately log normal.

The probability that a mean dose of $c$ cells will infect a host in a system of sensitivity $\alpha$ is—

$$1 - e^{-c\alpha} \tag{1}$$

If the sensitivities are distributed with probability density $f(\alpha)$, then the probability of infection of a host selected at random is

$$P = \int_0^1 (1 - e^{-c\alpha}) f(\alpha)\, d\alpha$$

$$= \int_{-\infty}^{0} (1 - e^{-e^{x+y}})\, \varphi(y)\, dy \tag{2}$$

where $x = \log c$, $y = \log \alpha$, $\varphi(y)\, dy = f(\alpha)\, d\alpha$

Define $\varphi(y) = 0$ for $y > 0$ and

$$P = \int_{-\infty}^{\infty} (1 - e^{-e^{x+y}})\, \varphi(y)\, dy \tag{3}$$

One can see that this is the distribution function of a variate

$$x = z - y \tag{4}$$

where $z$ is a variate distributed independently of $y$ with a distribution function

$$1 - e^{-e^{z}} \tag{5}$$

From (4) and independence of $z$ and $y$, we infer that the mean $E[x]$ and variance $V(x)$ are given by

$$E[x] = E[z] - E[y]$$
$$V[x] = V[z] + V[y]$$

hence

$$E[y] = E[z] - E[x]$$
$$V[y] = V(x) - V(z)$$

Irwin (1942) has studied the distribution of $z$ and shown that

$$E[z] = -\gamma = -0{\cdot}577$$

$$V(z) = \pi^2/6 = 1{\cdot}645$$

From the fitted probit line we have the estimates for

$$E[x] : \log (2 \times 10^6) - 11{\cdot}2 \log_e 2 = 6{\cdot}745$$

$$V(x) : 23{\cdot}15\, (\log_e 2)^2 = 11{\cdot}123$$

Hence the mean and variance of the distribution of the log sensitivities $y$ are estimated to be

$$E[y] : -7{\cdot}322$$
$$V(y) : \quad 9{\cdot}478 \qquad\qquad S.D.\ \ 3{\cdot}079$$

Since the probit line fits the data, $x$ is approximately normally distributed. $z$ is not too far from normal either, and in any case, $V(z)$ is only a small component of $V(x)$. Thus $y$ must be approximately normal, i.e. we have shown that the distribution of the log sensitivities is approximately normal, as mentioned earlier.

Our results may be summarized by plotting the distribution of sensitivities (Fig. 3). For convenience we use a scale of common logarithms, i.e. we plot

FIG. 3.—Frequency distribution of host sensitivities.
——— = estimated from the data.
– – – – – – = by extrapolation.

$\log_{10}\alpha = y/\log_e 10 = \cdot 434\, y$. $E[\log_{10}\alpha] = -3{\cdot}13$, $S.D.$ $(\log_{10}\alpha) = 1{\cdot}337$. The distribution should be truncated at $y = 0$, i.e. at $\alpha = 1$ because we defined that $\varphi(y) = 0$ for $y > 0$. As the truncation is slight it makes no appreciable difference to our estimates of the mean and variance.

Since only the high dosage levels were tested, only the upper half of the dose-response curve is established. This corresponds to the lower half of the distribution of $h$ involving those individuals which need large doses for any reasonable chance of infection. It follows that the lower half of the distribution of $h$ is well established by the data but the upper half (shown with dots in Fig. 3) is merely an extrapolation based on the normal fit.

If however, we consider the situation described under ($f$) in the theoretical introduction and we replace the formula (1) for the probability of infection by

$$1 - e^{-c\alpha}\,[1 + c\alpha + (c\alpha)^2/2! + \ldots + (c\alpha)^{g-1}/(g-1)!] \qquad (6)$$

the theory is the same except that the mean and variance of $z$ is now

$$E[z] = -\gamma + (1 + 1/2 + \ldots + 1/(g-1))$$

$$V[z] = \pi^2/6 - (1 + 1/2^2 + 1/3^2 + \ldots + 1/(g-1)^2)$$

In this model the variance of $z$ is even smaller and thus if it were applicable instead of (1), $y$ would still be approximately normal.

From our results we can conclude that about 850 cells are required to produce tumours in 50 per cent of animals. This is of the same order as Hewitt (1953) found with subcutaneous inoculations of Sarcoma 37 tumour cells. Also, it is plain from our results, that between one million and two million cells are required to produce tumours in 99 per cent of animals.

Our experiments did not cover the lower end of the dose-response curve, but it is interesting to speculate that, if the distribution remained the same throughout, it suggests that one cell would produce tumours in about one to two per cent of our animals. This would not be at variance with the findings of Hauschka (1953) who succeeded in producing ascites tumours in about 15 per cent of the more susceptible suckling mice following the inoculation of single Ehrlich and Krebs ascites tumour cells. He attributed his proportion of failures only to the lack of viability of his inoculated cells but it seems probable that host resistance was, at least in part, a factor.

Another point of interest is the wide variation in sensitivities. About 850 cells are required to infect 50 per cent of mice while about two thousand times as many are necessary to infect nearly all mice. If we assume that the curve may be extrapolated towards its lower end we find that 1 to 2 per cent of mice would be infected by a single cell. It is possible to suggest that genetic incompatibility between tumour and host is responsible for the widespread variation in sensitivities. However, it is interesting to compare our results with those of Hewitt (1953). With the number of cells expressed as common logarithms (see Hewitt's Fig. 1) the standard deviation for his distribution is approximately 1·6 and ours expressed in the same units, is remarkably close to it being 1.45. Hewitt's Fig. 1 shows that the standard deviations of the distribution of sensitivities of C3H mice inoculated with C3H sarcoma is very close to that of Sarcoma 37 and our own. Hence, using the standard deviation of the distribution of sensitivities as a measure, the quoted evidence suggests that considerable variation is characteristic of transplantable mouse tumours and does not appear to be diminished by genetic homogeneity.

The similarity of Hewitt's (1953) results with Sarcoma 37 and ours with the the Ehrlich tumour is remarkable. Although the two tumours exhibit well-known differences, another similarity between them is that neither tumour is discriminating in that each appears to grow in a wide variety of white mice. Both these tumours are distinguished from the C3H sarcoma grown in the strain of origin by having a larger ED 50, but show no marked difference in variability.

When we consider the dose-response relationships of ascites tumours only (Table I and Fig. 1) we see that it is similar to that for all tumours but are shifted to the right. This suggests that, on the average, a greater number of cells are required to produce ascites than tissue growth only. In our experience well-developed Ehrlich ascites tumours always have tissue growth in addition. These findings give us no reason to think otherwise than that the production of ascites follows tissue growth if the right conditions are present, and as we have shown occurs with Sarcoma 37 ascites tumour (Warner, Kroeker and Lederman, 1957).

Fig. 2 shows the distribution of the ratios of definite ascites tumours to non-ascites tumours. One might have expected the ratio to have remained constant where less than 100 per cent of animals respond with tumour. However, this is obviously not so unless our results represent a freak of chance. We have no simple explanation for this unexpected distribution. However, we offer the following tentative and obviously speculative hypothesis.

Let us assume that the development of ascites tumours or solid tumours depends on two factors : firstly, the number of inoculated tumour cells that establish themselves in tissues and, secondly, the rate of spread of those tumour cells where rapid spreading results in the production of ascites. Also let us assume that a high degree of susceptibility in mice is associated with the ability of tumour cells to spread rapidly in such mice. Although such characteristics would be subject to a continuous distribution among the mice, the hypothesis can be illustrated in Table IV where we consider high, medium and low doses of tumour cells,

TABLE IV.—*Illustration of Hypothesis to Explain Distribution of Ratios of Ascites to Non-Ascites Tumours*

| Dose | Definite ascites | Other tumours | No tumours |
|---|---|---|---|
| High | Intermediate and susceptible | Resistant | — |
| Medium | Susceptible | Intermediate | Resistant |
| Low | Susceptible | — | Resistant and intermediate |

resistant, susceptible and intermediate mice which develop definite ascites tumours, other tumours, or no tumours.

The high proportion of ascites tumours at high dosages is due to the large number of inoculated cells. Where the dose is large enough practically all mice develop ascites tumours. When the dose is diminished all mice develop tumours but the resistant ones fail to develop ascites tumours. With medium sized doses the susceptible mice develop definite ascites, the intermediate other tumours and the resistant no tumour at all. Low doses fail to cause any kind of tumours in resistant and intermediate animals—those susceptible mice that are infected are so susceptible that, once infected, they will allow rapid spread of tumour and hence the development of ascites. This would account for the high proportion of ascites tumours following small doses of tumour cells. This explanation finds some support from the work of Hauschka (1953) who found that, following the inoculation of single cells into suckling mice, all tumours that developed were ascites tumours.

### SUMMARY

The dose-response curve of the Ehrlich ascites tumour in mice was investigated in an attempt to learn something of the viability of tumour cells and the susceptibility of the hosts. The theoretical background of such experiments is discussed.

Six experiments were carried out in which serial dilutions of a suspension of tumour cells were inoculated intraperitoneally into mice. Observations were obtained on a total of 614 mice of which 386 developed typical ascites tumours and 129 other types of tumour growth.

The results were subjected to probit analysis from which it was concluded that approximately 850 tumour cells would produce tumours in 50 per cent of animals and that host susceptibility in the strain of mice used was variable. Consequently, no conclusion could be drawn concerning the viability of the tumour cells. However, it was possible to determine the distribution of the log " sensitivities " of the host-cell system where " sensitivity " is the probability of infection of an animal by a single cell. It was noted that this distribution was normal with considerable variance and that its parameters were apparently in fairly close agreement with those found for Sarcoma 37 by another author. Also the variance of these tumours was of the same order as a C3H tumour grown in the strain of origin although their ED 50 was considerably greater. It was concluded that sensitivity of these transplantable mouse tumours showed considerable variation which did not appear to be diminished by genetic homogeneity.

The distribution of the proportions of mice developing ascites tumours did not appear to be markedly different from that for all types of tumours apart from a shift to the right.

The ratio of ascites tumours to those without ascites exhibited a curious distribution when plotted against doses of cells. It was high with high doses, fell with intermediate doses and then rose again with very low doses. An attempt was made to provide an explanation for this phenomenon.

It is a pleasure to acknowledge the painstaking technical assistance of Brian Moore.

### REFERENCES

ARMITAGE, P. AND SPICER, C. C.—(1956) *J. Hyg., Camb.*, **54**, 401.
FINNEY, D. J.—(1952) ' Probit Analysis ', Second edition, Cambridge (University Press).
HALDANE, J. B. S.—(1939) *J. Hyg., Camb.*, **39**, 289.
HAUSCHKA, T. S.—(1953) *Trans. N.Y. Acad. Sci.*, **11**, 16, 64.
HEWITT, H. B.—(1953). *Brit. J. Cancer*, **7**, 367.
HOSKINS, J. M., MEYNELL, G. G. AND SANDERS, F. K.—(1956) *Exp. Cell Res.*, **11**, 297.
IRWIN, J. O.—(1942) *J. Hyg., Camb.*, **42**, 328.
ISAACS, A.—(1957) " Particle Counts and Infectivity Titrations for Animal Viruses," in ' Advances in Virus Research.' New York (Academic Press Inc.) Vol. IV, pp. 112–155.
WARNER, P., KROEKER, H. AND LEDERMAN, J. M.—(1957) *Brit. J. Cancer*, **11**, 93.